# Anti-tumour activity and toxicity of the new prodrug9-aminocamptothecin glucuronide (9ACG) in mice

**DOI:** 10.1038/sj.bjc.6600317

**Published:** 2002-05-03

**Authors:** Z M Prijovich, B-M Chen, Y-L Leu, J-W Chern, S R Roffler

**Affiliations:** Institute of Biomedical Sciences, Academia Sinica, Taipei, Taiwan 115; ICTM, Center for Chemistry, Belgrade, Yugoslavia; School of Pharmacy, National Taiwan University, Taipei, Taiwan; Chia-Nan College of Pharmacy and Science, Tainan Hsien, Taiwan

**Keywords:** 9-aminocamptothecin, glucuronide, toxicity, cancer, prodrug, topoisomerase I

## Abstract

Cancer chemotherapy is limited by the modest therapeutic index of most antineoplastic drugs. Some glucuronide prodrugs may display selective anti-tumour activity against tumours that accumulate β-glucuronidase. We examined the toxicity and anti-tumour activity of 9-aminocamptothecin glucuronide, a new glucuronide prodrug of 9-aminocamptothecin, to evaluate its potential clinical utility. 9-aminocamptothecin glucuronide was 25–60 times less toxic than 9-aminocamptothecin to five human cancer cell lines. β-glucuronidase activated 9-aminocamptothecin glucuronide to produce similar cell killing as 9-aminocamptothecin or topotecan. The *in vivo* toxicity of 9-aminocamptothecin glucuronide in BALB/c mice was dose-, route-, sex- and age-dependent. 9-aminocamptothecin glucuronide was significantly less toxic to female than to male mice but the difference decreased with age. 9-aminocamptothecin glucuronide and 9-aminocamptothecin produced similar inhibition (∼80%) of LS174T human colorectal carcinoma tumours. 9-aminocamptothecin glucuronide cured a high percentage of CL1-5 human lung cancer xenografts with efficacy that was similar to or greater than 9-aminocamptothecin, irinotecan and topotecan. The potent anti-tumour activity of 9-aminocamptothecin glucuronide suggests that this prodrug should be further evaluated for cancer treatment.

*British Journal of Cancer* (2002) **86**, 1634–1638. DOI: 10.1038/sj/bjc/6600317 www.bjcancer.com

© 2002 Cancer Research UK

## 

20(S)-camptothecin, an alkaloid isolated from *Camptotheca acuminata* (Wall *et al*, 1966), exhibits potent antineoplastic activity (Giovanella *et al*, 1989) by inhibition of topoisomerase I (Hsiang *et al*, 1985). Unfortunately, camptothecin is difficult to formulate due to its low solubility in water. In addition, its soluble sodium salt can produce unpredictable toxicity (Gottlieb *et al*, 1970). Water-soluble derivatives of camptothecin have been synthesised to overcome these problems and two such analogues (topotecan and irinotecan) are approved for clinical use (Coleman and Miller, 1997; Shimada, 1998). Clinical use of another analogue, 9-aminocamptothecin (9AC), is hindered by its low solubility in water (Herben *et al*, 1999) despite potent anti-tumour activity (Giovanella *et al*, 1996).

Glucuronides are generally less toxic and more water soluble than the parent compounds. These properties of glucuronides, together with the relatively high β-glucuronidase (βG, E.C.3.2.1.31) activities found in some cancers (Pearson *et al*, 1989; Albin *et al*, 1993), suggests that glucuronide prodrugs may be useful for cancer treatment. Although several normal tissues such as liver, spleen, colon and bone marrow contain high activities of βG, this enzyme is located in lysosomes (Price and Dance, 1967) and is therefore largely inaccessible to highly hydrophilic glucuronides which pass poorly through the cell membrane (Haisma *et al*, 1992). βG, however, may be more accessible in tumours (Bosslet *et al*, 1998), which combined with the acidic pH of the interstitial space (Tannock and Rotin, 1989), could allow preferential activation of glucuronide prodrugs in tumours. In fact, glucuronide metabolites of alkylating agents displayed potent anti-tumour activity in select tumour models (Connors and Whisson, 1966) but subsequent attempts to exploit differences in β-glucuronidase activity between tumours and normal tissues were largely unsuccessful for alkylating agents (Young *et al*, 1976). More recently, a glucuronide prodrug of doxorubicin has shown promising activity against experimental cancers (Houba *et al*, 2001). Tumour-selective antibodies have also been employed to deliver βG to tumours in a strategy termed antibody-directed enzyme prodrug therapy (ADEPT) (Bagshawe *et al*, 1988). Glucuronide prodrugs have displayed excellent anti-tumour activity when coupled with ADEPT (Bosslet *et al*, 1994), even for glucuronide prodrugs of alkylating agents that possess no activity by themselves (Chen *et al*, 1997).

We recently synthesised 9-aminocamptothecin glucuronide (9ACG), a prodrug in which glucuronic acid is connected via a self-immolative carbamate linker to the N9 atom of 9AC (Leu *et al*, 1999). 9ACG is water soluble, stable in human serum and is a substrate for βG (Leu *et al*, 1999). The aim of the present study was to investigate the toxicity and anti-tumour activity of 9ACG to help evaluate the potential of 9ACG as a soluble prodrug of 9AC for cancer monotherapy and ADEPT.

## MATERIALS AND METHODS

### Experimental animals

Male and female BALB/c and BALB/*c* *nu/nu* mice aged 5 to 20 weeks were allowed free access to food and water. All animal experiments were carried out with ethical committee approval. The ethical guidelines that were followed met the standards required by the UKCCCR Guidelines for the Welfare of Animals in Experimental Neoplasia (Workman *et al*, 1998).

### Cell lines

LS174T colon, AGS stomach, MCF-7 breast and OVCAR-3 ovarian human cancer cell lines were obtained from the American Type Culture Collection (Manassas, VA, USA). CL1-5 human lung adenocarcinoma cells (Yang *et al*, 1992) were kindly provided by Dr PC Yang, Department of Internal Medicine, National Taiwan University Hospital (Taipei, Taiwan). LS174T, CL1-5 and AGS cells were cultured in Dulbecco's modified Eagle's medium (Sigma, St Louis, MO, USA) whereas MCF-7 and OVCAR-3 cells were grown in RPMI-1640 (Sigma, St. Louis, MO, USA) supplemented with 10 μg ml^−1^ bovine insulin. All media were supplemented with 10% heat-inactivated bovine calf serum, 100 U ml^−1^ penicillin and 100 μg ml^−1^ streptomycin in a 5% CO_2_ humidified atmosphere.

### *In vitro* cytotoxicity

Five thousand (10 000 for MCF-7) cells per well were plated in 96 well microtiter plates overnight. Graded amounts of 9AC, topotecan, 9ACG or 9ACG plus 1 μg *E coli*-derived βG were added for 24 h in triplicate. The cells were then washed twice with sterile PBS and fresh medium was added for another 24 h. Fresh medium containing 1 μC per well [^3^H]thymidine was added 16 h before the cells were harvested on glass-fibre filters and the radioactivity was measured on a Topcount scintillation counter. The results are expressed as % inhibition=(c.p.m._S_×100)/c.p.m._C_ where c.p.m._i_ represents counts per minute of sample (S) or untreated controls (C).

### *In vivo* toxicity

9ACG (10 mg ml^−1^ in PBS) was adjusted to pH 6.5 with sodium carbonate immediately before administration. More than 95% of 9ACG remained in the lactone form for at least 4 h as determined by HPLC (data not shown). 9AC was prepared as a 1 mg ml^−1^ suspension in Lipiodol ultra-fluide (Laboratorie Guerbet, Bois Cedex, France). Mice were i.p. injected with 50 mg kg^−1^ 9ACG, i.v. injected with 25 or 50 mg kg^−1^ 9ACG or s.c. injected with 2.5 or 5 mg kg^−1^ 9AC in pilot studies using two mice per group. A maximum transient (<72 h) weight loss of less than 25% was considered acceptable. Six 7–8 week old mice (three males and three females) were also i.v. injected with 50 mg kg^−1^ 9ACG. Prodrug toxicity was further examined in three groups of six mice (three males and three females) at three different ages (5, 10 and 20 weeks). Mice were injected with 9ACG (50 mg kg^−1^, i.v.) or 9AC (5 mg kg^−1^, s.c.) and body weights were followed. Initial body weight was defined as the mean value of three determinations taken 4, 2 and 0 days before drug administration.

### *In vivo* antitumour activity

Two×10^6^ LS174T cells or 1×10^7^ CL1-5 cells were s.c. injected in the right flank of 16 week old female BALB/*c* *nu/nu* mice. Tumour bearing mice were subdivided into groups of five to eight mice. Therapy was initiated 10–11 days after tumour inoculation, when the mean tumour volume was 100–200 mm^3^. Mice treated with prodrug were i.v. injected with 9ACG at the indicated doses and times. The 9AC group was s.c. injected on the contralateral flank with 3 or 5 mg kg^−1^ 9AC as a suspension in lipiodol. Irinotecan (10 mg kg^−1^) and topotecan (1.8 mg kg^−1^) were i.v. injected on the indicated days. Control mice were i.v. injected with PBS following the corresponding 9ACG schedule. Tumour volumes were calculated as length×width×thickness×0.5 and expressed in mm^3^. Tumour growth inhibition was calculated as:


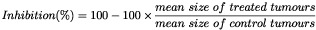


### Statistical analysis

Statistical significance of differences between mean values was estimated with Microsoft Excel using the independent *t*-test for unequal variances.

## RESULTS

### *In vitro* cytotoxicity

Table 1

**Table 1:**
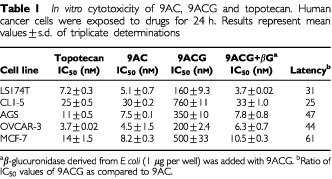
*In vitro* cytotoxicity of 9AC, 9ACG and topotecan. Human cancer cells were exposed to drugs for 24 h. Results represent mean values±s.d. of triplicate determinations

compares the *in vitro* cytotoxicity of topotecan, 9AC, 9ACG and 9ACG mixed with βG to five human cancer cell lines. Topotecan and 9AC displayed similar potencies with IC_50_ values ranging from 3 to 30 nM. 9ACG was 25 to 60 times less toxic than 9AC but displayed similar cytotoxicity as 9AC after the glucuronide group was enzymatically cleaved by βG.

### *In vivo* toxicity

**Figure 1:**
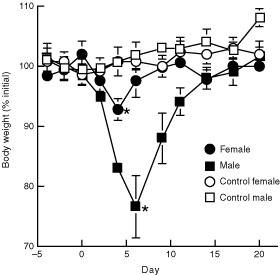
Toxicity of 9ACG. A single i.v. injection of 50 mg kg^-1^ 9ACG was given to male and female 7–8 weeks old BALB/c mice. Control male and female mice were untreated. Mean weights of three mice relative to initial body weights are shown. Significant differences between prodrug and control groups at weight nadirs are indicated: *P⩽0.05. Bars, s.e.

A single s.c. injection of 2.5 mg kg^−1^ 9AC suspended in lipiodol caused minimal toxicity with 10% weight loss whereas a dose of 5 mg kg^−1^ produced about 20% weight loss (results not shown). Mice that were i.p. injected with 50 mg kg^−1^ 9ACG experienced progressive weight loss and died within 6 days (results not shown). In contrast, all mice that were i.v. injected with 25 or 50 mg kg^−1^ 9ACG experienced dose-dependent weight loss until day 6 followed by rapid and complete recovery within a week. A more detailed study of toxicity performed in 7–8 week old male and female mice with 50 mg kg^−1^ 9ACG (Figure 1

) confirmed the preliminary results. Male mice transiently lost about 25% body mass but female mice experienced only about 5% weight loss.

**Figure 2:**
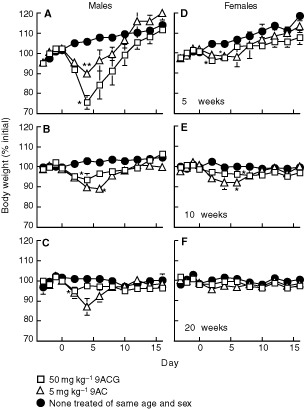
Influence of age and sex on 9ACG toxicity. Male (**A**–**C**) and female (**D**–**F**) mice aged 5 weeks (**A**, **D**), 10 weeks (**B**, **E**) or 20 weeks (**C**, **F**) were i.v. injected with 50 mg kg^−1^ 9ACG or s.c. injected with 5 mg kg^−1^ 9AC. Body mass was followed and compared to non-treated animals of the same age and sex. Mean weights of three mice relative to initial body weights are shown. Significant differences between drug and control groups at weight nadirs are indicated: *P⩽0.05; **P⩽0.005. Bars, s.e.

The effect of age and sex on 9ACG toxicity was further examined by injecting 5, 10 and 20-week old male and female mice with 9AC or 9ACG. An i.v. injection of 50 mg kg^−1^ 9ACG produced about 25% weight loss in 5-week old male mice (Figure 2A

), but only 5% weight loss in 20-week old mice (Figure 2C). 9ACG produced minimal (5%) toxicity in 5-week old female mice (Figure 2D) and virtually no weight loss in 10 or 20-week old mice (Figures 2E,F). Regardless of age, 5 mg kg^−1^ 9AC was more toxic to male mice, producing about 10% weight loss (Figures 2A,B,C) compared with 5% weight loss in female mice (Figures 2D,E,F).

### Therapy of human cancer xenografts

**Figure 3:**
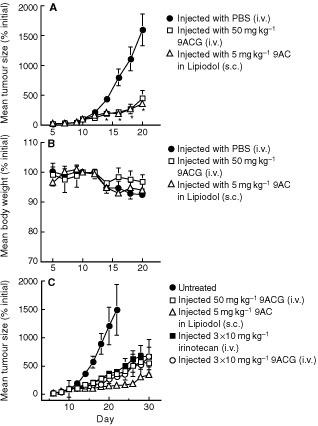
Anti-tumour activity of 9ACG against LS174T xenografts. (**A**, **B**) BALB/*c* *nu/nu* mice bearing LS174T tumours were i.v. injected with PBS, i.v. injected with 50 mg kg^−1^ 9ACG or s.c. injected with 3 mg kg^−1^ 9AC in Lipiodol on day 10. Mean tumour sizes (**A**) and weights (**B**) of six mice relative to mean values at the initiation of therapy (day 10) are shown. The mean size of drug-treated (9AC and 9ACG) tumours was significantly (*P*⩽0.05) smaller than control tumours after day 13. (**C**) BALB/*c* *nu/nu* mice bearing LS174T tumours were untreated, i.v. injected with 50 mg kg^−1^ 9ACG or s.c. injected with 5 mg kg^−1^ 9AC in Lipiodol on day 10, or i.v. injected with 10 mg kg^−1^ 9ACG or irinotecan on days 10, 12 and 14. Results represent mean tumour size of 7–8 mice relative to mean tumour size on day 10. The mean size of drug-treated (9AC, 9ACG and irinotecan) tumours was significantly (*P*⩽0.05) smaller than control tumours after day 12. Bars, s.e.

BALB/*c nu/nu* mice bearing established LS174T tumours were i.v. injected with 50 mg kg^−1^ 9ACG in PBS or s.c. injected with 3 mg kg^−1^ 9AC as a suspension in lipiodol. Both 9ACG and 9AC inhibited LS174T tumour growth by about 80% (Figure 3A

). Neither 9ACG nor 9AC produced significant weight loss in this experiment (Figure 3B). To investigate the influence of dose and schedule on the anti-tumour activity of 9ACG, BALB/*c nu/nu* mice bearing LS174T xenografts were i.v. injected once with 50 mg kg^−1^ 9ACG or three times with 10 mg kg^−1^ 9ACG. Tumour-bearing mice were also i.v. injected three times with 10 mg kg^−1^ irinotecan or s.c. injected once with 5 mg kg^−1^ 9AC. The fractionated 9ACG schedule, despite a lower total dose, produced similar anti-tumour activity as one bolus injection of 9ACG (Figure 3C). The anti-tumour activity produced by 9ACG and CPT−11 was similar. 9AC produced good anti-tumour activity but was unexpectedly toxic, killing three of six mice by day 8. Although 9ACG and CPT-11 produced similar maximum weight loss of about 20%, one of six mice died after treatment with 50 mg kg^−1^ 9ACG or three fractionated doses of 10 mg kg^−1^ 9ACG.

**Figure 4:**
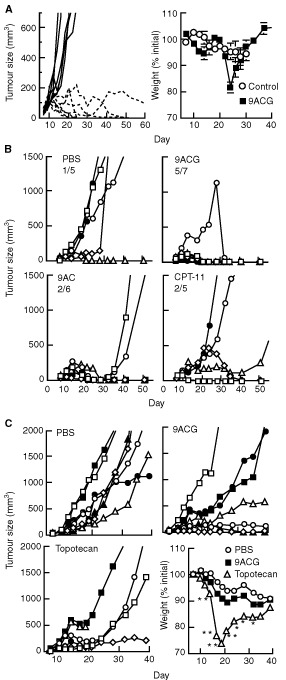
Anti-tumour activity of 9ACG against CL1-5 human lung adenocarcinoma xenografts. (**A**) BALB/*c* *nu/nu* mice implanted with CL1-5 tumour cells on day 0 were i.v. injected with PBS (solid lines) or 50 mg kg^−1^ 9ACG (dashed lines) on days 10 and 20. Results show the tumour sizes of individual mice. Mean body weights of control or 9ACG-treated mice are shown relative to mean weights on day 5. Bars, s.e. (**B**) Nude mice implanted with CL1-5 cells on day 0 were i.v. injected with PBS (PBS) or 50 mg kg^−1^ 9ACG (9ACG) on day 10, s.c. injected with 3 mg kg^−1^ 9AC in Lipiodol on day 10 (9AC), or i.v. injected with 10 mg kg^−1^ irinotecan on days 10, 12 and 14 (CPT-11). The ratio of long-term survivors (>120 days tumour-free) in each group is indicated. (**C**) Nude mice implanted with CL1-5 cells on day 0 received i.v. injections of PBS (PBS) or 8 mg kg^−1^ 9ACG (9ACG) on days 11, 13, 15, 24, 26 and 28 or 1.8 mg kg^−1^ topotecan on days 11, 12, 13, 14 and 15 (topotecan). Mean body weights of the PBS, 9ACG or topotecan treated mice are shown relative to mean weights on day 8. Significant differences between the mean weight of mice treated with topotecan or PBS are indicated: *P⩽0.05; **P⩽0.0005. Error bars are not shown for clarity.

Intravenous administration of 50 mg kg^−1^ 9ACG on days 10 and 20 resulted in complete regression of six of seven CL1-5 tumours (Figure 4A

). The treated mice experienced 14% weight loss after the second prodrug injection, but all the mice quickly recovered and long-term observation (100 days after therapy) did not reveal any signs of delayed toxicity. Figure 4B compares the treatment of CL1-5 tumours by 9ACG, 9AC or irinotecan. 9AC significantly (*P*⩽0.05) suppressed tumour growth but four of six mice experienced tumour relapse within 100 days. Irinotecan produced long-term tumour regression in two of five mice but tumour size was not significantly delayed as compared to PBS-treated control mice. One i.v. injection of 50 mg kg^−1^ 9ACG significantly (*P*⩽0.05) suppressed tumour growth; five of seven mice were apparently cured and tumour growth was suppressed in another mouse for over 100 days. One mouse died after prodrug treatment indicating toxicity although the maximum mean weight loss was only 7.5% after prodrug treatment. One of five control tumours failed to grow in this experiment. Figure 4C compares the treatment of CL1-5 tumours with fractionated doses of 9ACG or topotecan. Treatment with six injections of 8 mg kg^−1^ 9ACG produced significant (*P*⩽0.05) delay of tumour growth and tumour regressions in three of seven mice. A previously described optimal schedule for topotecan (Zamboni *et al*, 1998) displayed minimal anti-tumour activity against CL1-5 xenografts and was highly toxic; two of six mice died and mean mouse weight was significantly (*P*⩽0.0005) decreased as compared with control mice. The weight of mice treated with 9ACG did not significantly differ from control mice in this experiment.

## DISCUSSION

Glucuronide analogues of anti-neoplastic drugs offer the possibility of increasing treatment specificity for tumours that accumulate βG (Bosslet *et al*, 1998; Houba *et al*, 1998, 2001). Potential prodrugs must exhibit reduced toxicity compared to the parent drug as well as allow specific conversion to the parental drug by βG. 9ACG was less toxic than 9AC to all the cells examined, suggesting that the glucuronide group of 9ACG hindered diffusion of the highly lipophilic 9AC moiety through the cell membrane. Addition of βG, however, increased the cytotoxicity of the prodrug to similar levels as 9AC, showing that 9ACG was converted to parent drug. 9ACG thus behaves as a glucuronide prodrug.

The toxicity of 9ACG in mice depended on their sex and age; 9ACG was less toxic to female and to older mice. βG is induced in mice in the epithelial cells of the kidney proximal tubule by androgen (Paigen, 1989). Thus, βG activity is higher in male mice (Johnson *et al*, 1986). Consistent with this, a glucuronide prodrug of doxorubicin was also more toxic to male mice (Bosslet *et al*, 1998). βG is not induced by androgen in humans (Paigen, 1989), suggesting that glucuronide prodrugs may not display gender-related differential toxicity in cancer patients. 9ACG was also less toxic to older mice. This was not due to differences in topoisomerase I activity, a determinant of camptothecin cytotoxicity, because 9AC toxicity was independent of age (Figure 2). βG activity may decrease with age (Traurig, 1976), suggesting that activation of 9ACG by endogenous βG may be responsible for prodrug toxicity in young mice. The high toxicity of 9ACG after i.p. administration demonstrated that prodrug toxicity also depended on the route of administration. The mechanism of increased prodrug toxicity after i.p. injection is currently unknown.

9ACG displayed good anti-tumour activity against LS174T human colon cancer xenografts in nude mice with acceptable levels of toxicity. Treatment of tumour-bearing mice with 9ACG produced similar inhibition of tumour growth as irinotecan. In addition, 9ACG was equally effective but produced less toxicity than 9AC. 9ACG was even more effective against CL1-5 human lung adenocarcinoma tumours. 9ACG cured 70–85% of mice with established CL1-5 tumours. 9ACG appeared to be more effective than 9AC or topotecan against CL1-5 tumours. Topotecan produced poor anti-tumour activity at an optimal dose schedule (Zamboni *et al*, 1998) and was unacceptably toxic. The superior anti-tumour activity of 9ACG to CL1-5 tumours compared with LS174T tumours could not be predicted by the *in vitro* sensitivity of these tumour cells to 9AC or 9ACG (Table 1), indicating that other factors, such as local *in vivo* tumour pH or βG levels are likely to be important determinants of tumour sensitivity to 9ACG.

9ACG was originally synthesised as a prodrug for antibody-directed enzyme prodrug therapy (ADEPT). In this treatment strategy, prodrugs are selectively activated at tumour cells that have been pre-targeted with antibody-enzyme conjugates (immunoenzymes). The finding that 9ACG produced anti-tumour activity without the need for immunoenzyme targeting suggests that sufficient βG is present at tumours to convert 9ACG to 9AC. This contrasts with a glucuronide prodrug of p-hydroxyaniline mustard (BHAMG), which only displays anti-tumour activity when tumours are pre-targeted with βG immunoenzymes (Chen *et al*, 1997). Some tumours express high levels of βG (Pearson *et al*, 1989; Albin *et al*, 1993) and βG may also be secreted by monocytes and granulocytes in necrotic areas of tumours (Bosslet *et al*, 1998). Extracellular βG effectively activated a glucuronide prodrug of doxorubicin in solid tumours (Bosslet *et al*, 1998), suggesting that a similar mechanism may be responsible for the *in vivo* activity of 9ACG. Because p-hydroxyaniline mustard is about 1000 times less cytotoxic than 9AC, it is likely that insufficient BHAMG is activated by endogenous βG to produce anti-tumour activity.

In summary, the toxicity of 9ACG in mice depended on sex, age, dose and route of administration. 9ACG displayed excellent anti-tumour activity against established human lung xenografts. The potent anti-tumour activity of 9ACG together with its high water solubility and stability (Leu *et al*, 1999) and potential tumour selectivity suggest that this prodrug shows promise for cancer therapy.
